# Successful outcomes of second hematopoietic stem cell transplantation for graft failure in pediatric patients with severe aplastic anemia

**DOI:** 10.1038/s41598-022-14665-1

**Published:** 2022-06-22

**Authors:** Meijie He, Ruirui Gui, Yingling Zu, Zhen Li, Dao Wang, Yanna Mao, Xianjing Wang, Huili Wang, Yongping Song, Jian Zhou

**Affiliations:** 1 Department of Hematology, Affiliated Cancer Hospital of Zhengzhou University and Henan Cancer Hospital, Zhengzhou, 450003 Henan China; 2 The First Affiliated Hospital of Zhengzhou University, Zhengzhou, 450003 Henan China; 3 Children’S Hospital Affiliated of Zhengzhou University and Henan Children’s Hospital, Zhengzhou, 450008 Henan China; 4 The Third People’s Hospital of Zhengzhou, Zhengzhou, 450000 Henan China

**Keywords:** Stem cells, Haematopoietic stem cells

## Abstract

Severe aplastic anemia (SAA) is a life-threatening hematological disorder. The major therapies include matched sibling donor (MSD)- hematopoietic stem cell transplantation (HSCT), matched unrelated donor (MUD)-HSCT and immunosuppressive therapy (IST). However, there are many problems that can occur after HSCT, and graft failure (GF) is one of the most serious complications. To find an effective treatment, we analyzed 10 cases of second HSCT to treat SAA pediatric patients who suffered from GF and concluded that second haploidentical family donors HSCT is an effective treatment. Moreover, adding a small dose of busulfan or 2 ~ 3 Gy total body irradiation (TBI) in nonmyeloablative regimens (NMAs) can promote the engraftment. Although the study also showed that PBSCs, as a source of stem cells, can promote the implantation of neutrophil cells, due to small sample size, more research is still needed.

## Introduction

Severe aplastic anemia (SAA) is a fatal disease that is caused by immune-mediated destruction of the hematopoietic progenitor cells and is characterized by a decrease in bone marrow cells and pancytopenia. According to the *Pediatric Amendment to Adult BSH Guidelines for Aplastic Anemia*, matched sibling donor (MSD)- hematopoietic stem cell transplantation (HSCT) is the first line treatment for SAA pediatric patients thus far^1^. We know that from 2008 to 2018, among the 1156 SAA patients who underwent MSD-HSCT, the 3-year overall survival rate was 93% ± 1% in the Chinese Blood and Marrow Transplantation Registry Group (CBMTRG).When there is a lack of a suitable MSD, immunosuppressive therapy (IST) and matched unrelated donor (MUD)-HSCT can also be first-line therapies. However, pediatric patients may also face many problems, such as graft failure (GF), relapse, acute or chronic graft versus host disease (GVHD) and other late effects after HSCT. The mortality rate caused by GF is approximately 3% to 16%^2^. IST before HSCT and ages beyond 15 years old are both risk factors that can increase the rate of GF^3^. A few cases have shown that patients who suffer from GF can also achieve good curative efficacy through MSD-HSCT or MUD-HSCT. However, only a few patients underwent haploidentical family donors HSCT. Therefore, we collected the information of 10 pediatric patients who experienced second HSCT (HSCT-2) after GF, some of whom underwent haploidentical-matched family donor-HSCT, aiming to provide a feasible treatment for pediatric patients who suffered from GF or relapse.

## Methods

We collected the information of 10 patients who suffered from GF after first HSCT (HSCT-1) from 1 May in 2004 to 10 March in 2021 in 4 centers. Among them, 5 were males and the others were females. The average age at diagnosis was 7 years old (ranging from 1.9 to 13.0 years, and the median age was 7 years old). According to the laboratory results and clinical data, patients who were diagnosed as congenital bone marrow disorders (Fanconi anemia, Diamond-Blackfan anemia, and dyskeratosis congenital (DKC)) were excluded. HLA compatibility was determined by high-resolution DNA techniques for the HLA-A, B, C, DRB1 and DQB1 loci before HSCT-2. Without HLA-MSD or HLA-MUD, HLA- haploidentical-matched family donors can be graft sources. The following conditioning regimens, including Fludarabine (Flu) + Cyclophosphamide (CTX) + anti-thymocyte protein (ATG) or anti-lymphocyte protein (ALG), were applied in all patients: Flu: 150 mg/m^2^,-6 ~ -2day; CTX: 100 mg/kg/day,-6 ~ -3 day; ATG 2 mg/kg/day,-4 ~ -1 day, or ALG:4 mg/kg/day; some of the patients also received a small dose of busulfan (Bu, 3.2 mg/kg/day), melphalan (Mel, 100 mg/m^2^/day) or total body irradiation (TBI). The doses of mononuclear cells (MNCs) were all over 5 × 10^8^/kg, and the doses of CD34^+^ cells were over 2 × 10^6^/kg. Low doses of PT-CY and mycophenolate mofetil (MMF) were used in all patients to prevent acute and chronic GVHD. Additionally, some patients also received Methotrexate (MTX).

Routine blood examinations were performed daily after transplantation to search for evidence of implantation. One month, 2 months and 3 months after the HSCT-2, patients were evaluated bone marrow chimerism to evaluate the status of bone marrow implantation. The time for neutrophil engraftment was defined as the first day when the neutrophil count is over 0.5 × 10^9^/L for 3 successive days after infusing stem cells. Primary GF was defined as follows: 28 days after infusion, the neutrophil count was lower than 0.5*10^9^/L. Secondary GF meant that after the partial or complete recovery of donor hematopoiesis, the neutrophil count was again lower than 0.5 × 10^9^/L.

The study was approved by the Ethical Committee of Affiliated Cancer Hospital of Zhengzhou University and Henan Cancer Hospital (Approval Number: 2019287) and was performed in accordance with relevant guidelines and regulations. All patients and their families agreed to the therapy and signed informed consent forms.

### Statistics

Descriptive statistical methods (such as the median, average, minimum, and maximum,) were used to assess the statistics. The end point of the research was death or GF. Each risk factor was analyzed by one-way analysis of variance. A P < 0.05 was considered statistically significant.

## Results

In our study, 10 patients’ data were collected. PNH status was evaluated before transplantation through FLAER, and none of them had been diagnosed as PNH. Cytogenetics characters were also tested, and the results were negative in all the patients. Before HSCT-1, only one patient received ATG and blood transfusion with poor efficacy. One patient only received blood transfusion before HSCT-1. And 8 of the 10 patients (including the patient who received ATG) received cyclosporin A (CsA) and blood transfusion. The last patient only used drugs which could promote hematopoiesis and blood transfusion. The average interval between the time of diagnosis and HSCT-1 was 14 months (ranging from a month to 56 months). There were 2 patients undergoing MSD-HSCT and 2 undergoing MUD-HSCT, and the others all used family-haploidentical donors. The conditioning regimen was FLU + CTX + ATG. Only one patient was infused with bone marrow stem cells (BMs) and peripheral blood stem cells (PBSCs). The others were all infused with PBSCs (Table 1). Low dose of PT-CY and MMF were used to prevent acute and chronic GVHD, and MTX was applied additionally in 3 patients. According to the standard of HCT-CI^4^, the scores of the patients were all 1. After HSCT-1, 4 patients were diagnosed with primary GF due to failing neutrophil engraftment or a chimerism rate lower than 30%. The other 6 patients were diagnosed with secondary GF due to neutrophil and platelet counts decreasing again, although neutrophils and platelets were engrafted. Once GF was diagnosed, while giving supportive treatment such as blood transfusion, various related examinations were performed as soon as possible to assess the condition of the recipients (ages and performance status) and the characteristics of the donors. After communicating with the families of patients, we decided to perform HSCT-2.

**Table 1:** The basic information of the pediatric patients of HSCT-1.

No	1	2	3	4	5	6	7	8	9	10
Sex	Male	Female	Female	Female	Male	Female	Male	Female	Male	Male
Age(years)	2.5	9.7	8.7	6.6	1.9	13	5.3	4	7.3	11.5
Treatment before HSCT-1	Stanozolol + VIT B12 + folic acid + Component blood transfusion	CsA + stanozolol + Component blood transfusion	Component blood transfusion	CsA + stanozolol + Component blood transfusion	CsA + Component blood transfusion	CsA + stanozolol + Danazol + Component blood transfusion	CsA + Component blood transfusion	CsA + stanozolol + ATG + Component blood transfusion	CsA + Component blood transfusion	CsA + Component blood transfusion
The internal between diagnosis and HSCT-1(months)	2	56	2	35	8	24	2	8	1	2
Donor of HSCT-1	MSD	haploidentical family donor	haploidentical family donor	haploidentical family donor	haploidentical family donor	MUD	haploidentical family donor	MUD	haploidentical family donor	MSD
Conditioning regimen	FLU + CTX + ATG	FLU + CTX + ATG	FLU + CTX + ATG	FLU + CTX + ATG	FLU + CTX + ATG	FLU + CTX + ATG	FLU + CTX + ATG	FLU + CTX + ATG	FLU + CTX + ATG	FLU + CTX + ATG
Graft source	UC	PBSC	PBSC	PBSC	PBSC	PBSC	PBSC + BM	PBSC	PBSC	PBSC
The dose of MNCs (*10^6^) and CD34^+^ (*10^6^) cells		MNC:9.63 CD34^+^ :5.82	MNC:9.8 CD34^+^ :6.3	MNC:11.92 CD34^+^ :5.96	MNC:14.65 CD34^+^ :6.59	MNC:7.6 CD34^+^ :5.6	BM: MNC:1.74 CD34^+^ :2.75 PBSC: MNC:6.51 CD34^+^ :5.84	MNC:7.39 CD34^+ ^:2.23	MNC:7.58 CD34^+ ^:10.6	MNC:9.16 CD34^+^ :6
Agents to prevent GVHD	A low dose of PT-CY + CsA + MMF	A low dose of PT-CY + CsA + MMF	A low dose of PT-CY + CsA + MMF	A low dose of PT-CY + CsA + MMF	A low dose of PT-CY + CsA + MMF	A low dose of PT-CY + CsA + MMF + MTX	A low dose of PT-CY + CsA + MMF	A low dose of PT-CY + CsA + MMF	A low dose of PT-CY + CsA + MMF	A low dose of PT-CY + CsA + MMF + MTX
Time of neutrophil engraftment	14	Failure in engraftment	Failure in engraftment	11	18	12	Failure in engraftment	13	Failure in engraftment	12
Time of platelet engraftment	14	Failure in engraftment	Failure in engraftment	12	Failure in engraftment	13	Failure in engraftment	17	Failure in engraftment	17
HCT-CI	1	1	1	1	1	1	1	1	1	1
Signs of GF	One year after hematopoiesis recovery, the count of blood cells decreased again	The chimerism rate was lower than 30%	The chimerism rate was lower than 30%	The count of blood cells was progressively decreasing	The count of blood cells was progressively decreasing	The count of blood cells and chimerism rate was progressively decreasing	The chimerism rate was lower than 30%	The count of blood cells and the chimerism rate was progressively decreasing	The count of blood cells was progressively decreasing	The count of blood cells and the chimerism rate was progressively decreasing
The type of GF	Secondary	Primary	Primary	Secondary	Secondary	Secondary	Primary	Secondary	Primary	Secondary

The interval between the two HSCT scans was 184 days (ranging from 19 to 1000 days, and the median time was 49 days). All primary GF patients experienced a second HSCT within 60 days. The graft sources included umbilical stem cells, PBSCs and bone BMs. (Table 2).

**Table 2:** The information of the pediatric patients of HSCT-2.

No	1	2	3	4	5	6	7	8	9	10
Internal of the two transplantations(days)	370	20	19	36	82	170	51	46	47	1000
Whether to change the donor	Yes	Yes	Yes	No	Yes	No	Yes	Yes	No	Yes
CMV of pre-transplantation	Negative	Negative	Negative	Negative	Negative	Negative	Negative	Negative	Negative	Negative
Donor	MSD	Haploidentical family donor	Haploidentical family donor	Haploidentical family donor	MUD	MSD	Haploidentical family donor	Haploidentical family donor	Haploidentical family donor	MUD
Conditioning regimen	CTX + ATG	TBI + FLU + CTX + ALG + BU	TBI + FLU + CTX + ALG + BU	TBI + FLU + CTX	TBI + BU + CTX + ALG	TBI + FLU + CTX	TBI + FLU + CTX + ALG + Mel	TBI + FLU + CTX + ATG	TBI + FLU + CTX + ATG	FLU + CTX + ATG
Graft resource	UC	BM + PBSC	BM + PBSC	PBSC	PBSC	PBSC	PBSC + UC	BM + PBSC + UC	BM + PBSC + UC	PBSC
The dose of MNCs (*10^6^) and CD34^+^ (*10^6^) cells	Unknown	PBSC:MNC:10.35 CD34^+ ^:6.29 BM: MNC:3.18 CD34^+^ :0.83	PBSC: MNC:11.17 CD34^+^ :5.8 BM: MNC:4.38 CD34^+^ :2.89	MNC:13.37 CD34^+^ :5.27	MNC:12.8 CD34^+ ^:8	MNC:8.8 CD34^+^ :2.45	MNC:6.8 CD34^+^ :5.27	PBSC: MNC:9.38 CD34^+^ :7.23 BM: MNC:3.5 CD34^+ ^:1.3	PBSC: MNC:8.3 CD34^+^ :6.5 BM: MNC:3.6 CD34^+^ :1.8	MNC:10.4 CD34^+ ^:5.6
Time of neutrophil engraftment	18	13	11	12	12	10	13	16	17	12
Time of platelet engraftment		14	26		17	14	20	19	52	25
Medicine of anti-GVHD	A low dose of PT-CY + CsA + MMF + MTX	A low dose of PT-CY + CsA + MMF	A low dose of PT-CY + CsA + MMF	A low dose of PT-CY + CsA + MMF	A low dose of PT-CY + CsA + MMF + MTX	A low dose of PT-CY + CsA + MMF	A low dose of PT-CY + CsA + MMF	A low dose of PT-CY + CsA + MMF	A low dose of PT-CY + CsA + MMF	A low dose of PT-CY + CsA + MMF + MTX
GVHD	Acute skin GVHD	Chronic liver GVHD		Acute intestine GVHD		Acute intestine GVHD	Acute skin and intestine GVHD		Acute skin and intestine GVHD	
CMV post-transplantation	Negative	Positive	Positive	Negative	Positive	Negative	Negative	Negative	Negative	Negative
Time of disease-free survival (days)	160	1000	1002	130	1011	98	128	1752	1753	191
Outcomes	The chimerism was completely donor type at one month, but the patient died of lung infection 5 months after HSCT-2	The chimerism was completely donor type at 32 months	The chimerism was completely donor type at 19 months	The chimerism was completely donor type at 5 months	The chimerism was completely donor type at 12 months	The chimerism rate was 94.86%	The chimerism was completely donor type at 2 months	The chimerism was completely donor type at 58 months	The chimerism was completely donor type at 58 months	The chimerism was completely donor type at 5 months

Among the 10 patients, 7 patients changed their donor. Among the 7 patients, 4 underwent family-haploidentical donor HSCT. Among the 3 patients who did not change donors, 2 were still using family-haploidentical donors. The average time for neutrophil engraftment in the 10 patients was 14 days, and the average time for platelet engraftment was 19 days. The average neutrophil engraftment times for children using haploidentical-matched family donors, MSDs, and MUDs were 14 days, 14 days, and 12 days, respectively. The average platelet engraftment times were 26 days, 14 days, and 21 days, respectively.

The conditioning regimen depended on the status of the patients. According to the Center for International Blood and Marrow Transplant Research (CIBMTR) guidelines, all patients received NMAs. To achieve the better efficacy, a low dose of TBI was applied in 8 patients, and 3 used a low dose of Bu, and only a patient used Mel. The 6 patients who used haploidentical-matched family donors, all received 2 ~ 3 Gy TBI, 2 of them used Bu, and only one patient used Mel. Among the 4 patients who used MSDs or MUDs, 2 patients received 2 ~ 3 Gy TBI. The average time of neutrophil engraftment was 13.0 days in the 8 patients (ranging from 10.0 days to 17.0 days). The average time of platelet engraftment was 23 days (ranging from 14.0 days to 52 days). In the other 2 patients, the average times of neutrophil and platelet engraftment were 15.0 days and 25.0 days, respectively.

With an average follow-up of 722.5 days (ranging from 98 to 1753 days), 9 patients were alive, and one patient died from lung infection after 5 months after HSCT-2. Including the deceased patient, a total of 3 patients were diagnosed with poor graft function (PGF) because frequent platelet transfusion was needed when excluding disease relapse, the influence of drugs, infection and other explanations^5^.

Low doses of PT-CY and MMF were used in all the 10 patients to prevent acute and chronic GVHD. Given that the graft resources were mainly from peripheral blood, to deplete the T lymphocytes in the stem cells, 3 patients additionally received MTX. However, 4 patients suffered from acute GVHD, including grade I-II acute skin, oral and/or intestinal GVHDs. Only one patient had chronic skin GVHD. In addition, tacrolimus was continually used to treat GVHD. Thirty days after HSCT-2, all patients were evaluated for donor chimerism, 9 patients showed full chimerism, and one patient showed mixed donor chimerism^5^. There was no patient suffering from GF after HSCT-2.

Apart from these, all the patients tested the copy number of cytomegalovirus (CMV) before and after transplantation. None of them were positive before HSCT-2 but there were 3 patients suffered from CMV reactivation. Foscarnet and human Immunoglobulin (pH4) were used to treat CMV infection. And the results turned to be negative after two weeks on average.

## Discussion

For SAA pediatric patients, HSCT should be performed as soon as possible if an MSD exists. If there is no MSD, the use of MUD-HSCT or IST should be decided according to whether there is a suitable donor, a graft resource, the patient’s status, the opinions of the pediatric patient’s family and so on. However, whether MSD-HSCT or MUD-HSCT is used, GF may occur and is one of the most serious complications after transplantation^6^. During 1998 and 2012, among 123 patients with HSCT in China, the incidence rate of GF was approximately 2.4%^7^. In Japan, from1992 to 2009, among 213 patients who underwent HSCT, the incidence rate of GF was approximately 5.6%^8^. Meanwhile, among 374 SAA patients who underwent MSD-HSCT in Europe, the rate was approximately 8%^9^. Only when the patients can recover autologous hematopoietic function will a second HSCT not be needed. According to the study from *the Aplastic Anemia Working Party of the European Group for Blood and Marrow Transplantation (WPSAA, EBMT)*, the probability of autologous hematopoietic function is only 4.2%^10^. Therefore, when GF occurs, we should estimate the status of the pediatric patients as soon as possible and perform HSCT-2 when necessary. In this study, we collected information on 10 pediatric patients with SAA, all of whom suffered from primary or secondary GF after HSCT-1. Although one patient died from lung infection 5 months after HSCT-2, neutrophils were implanted 14 days after transplantation, which suggested that a second transplantation after GF may have been a viable salvage therapy.

Although most patients can benefit from HSCT, the source of the graft is a major problem. A large sample size report from the Center for International Blood and Marrow Transplant Research (CIBMTR) indicated that the probabilities of primary GF or secondary GF at 100 days were up to 21% and 25%, respectively, after transplantation with 1- or > 1-antigen-mismatched related donors^11^. Over recent decades, great progress has been made in haplo-HSCT for hematological malignant disease due to the advent of novel conditioning regimens, optimized draft manipulation, improved GVHD prophylaxis, and advances in supportive care. A prospective study on HLA haplotype-mismatched family donor HSCT to treat hematological disease showed that donor chimerism was more rapidly achieved in patients who underwent HLA haploidentical-matched family donor HSCT than in those who underwent HLA-matched HSCT^12^. Some studies also thought that the matching degree of HLA (6/6 and 4/6 or 5/6) had no influence on implantation and survival time after the second transplantation using umbilical cord blood^13^. In our study, only 4 patients had MSD or MUD, and the other 6 patients underwent HLA haploidentical-matched family donor HSCT. After HSCT-2, all 6 pediatric patients achieved neutrophil engraftment, and the average time of engraftment was 14 days, which was slightly longer than the engraftment time of the Seung-Ah Yahng team (12.0 days)^14^, but significantly shorter than the engraftment time of the Yasushi Onishi team (24.5 days)^13^. Notably, both the Seung-Ah Yahng team and Yasushi Onishi team performed MSD-HSCT, and not all patients achieved neutrophil engraftment. What’s more, we collected 40 cases of pediatric SAA patients and 12 of them received haplo-HSCT. 3 of them suffered from GF, and one patient died from severe bacterial infection after engraftment. The others all achieved disease-free survival. These results suggested that haploidentical-matched family donor HSCT is an effective therapy for SAA patients. For SAA patients who suffer from GF after HSCT-1, it is an alternative option. Moreover, it is a considerable question whether to change donors. Previous studies have shown that there are no statistically significant differences in the prognosis of patients who change donors^15^. However, we think that for patients who suffer from secondary GF, if donor chimerism is over 50%, it is better not to change the donors. Therefore, in our study, when one pediatric patient with primary GF was rechecked for donor chimerism 30 days after HSCT-1, it was found that although neutrophils and platelets were not implanted, donor chimerism was up to 90%. Therefore, we still used the original donor.

In addition, conditioning regimens have a great influence on the efficacy of HSCT. There are some cases reports about SAA treated by haploidentical-matched family donor-HSCT, and the studies have shown that TBI-based conditioning for SAA demonstrated superiority. Although the numbers are too small to justify TBI-based conditioning for haploidentical-matched family donor-HSCT, we can also learn from their experience^15,16^. Moreover, studies have also shown that the use of Bu in conditioning regimen can reduce the risk of mixed chimerism after transplantation. So, considering that all 10 patients suffered from HSCT-1 failure, we added a small dose of Bu or TBI. We expected that the use of them could effectively promote the implantation of grafts and achieve long-term disease-free survival. Moreover, a recent study has shown that when low-dose irradiation or BU was added to conditioning regimens, the prognosis of transplantation from a 6–7/8 HLA-matched donor was comparable to that from an 8/8 HLA-matched unrelated donor in SAA patients under 40 years of age^17^. Führer et al. also believed that TBI can deplete not only most of the lymphocytes in bone marrow but also most of the lymphocytes in peripheral lymph nodes, which can reduce the recipients’ immune resistance and promote the engraftment of stem cells. It is especially suitable for patients with high frequency transfusion and lymphocytes involved in allergic sensitization^3^. The results showed that the average times of neutrophil and platelet engraftment of the 8 patients who used a low dose of TBI were 13.0 days and 23 days, respectively. Compared with the situation of patients who did not use TBI, the use of TBI did not lead to a prolonged engraftment time or increase the probability of acute or chronic GVHD.

Previous studies have shown that the mortality rate of pediatric patients with infused PBSCs is higher than that of patients with infused BMs^18^. However, we analyze the 40 cases who used PBMC as graft resources, only 2 of them died from severe bacteria or virus infection, 3 of them suffered from GF, others all achieved disease-free survival. Furthermore, we found that the use of PBSCs or BMs did not influence the probability of non-relapse-related mortality after HSCT-2 and acute or chronic GVHD. What’s more, the engraftment time of neutrophils in patients who only received infused PBSCs was shorter than that in patients who received infused PBSCs and BMs. Given the small sample size, we cannot conclude that the phenomenon is universal, and it needs to be verified in future research. Studies have also shown that the prognosis of primary GF is worse than that of secondary GF. This may be related to the shorter interval between the two HSCTs in primary GF. Because a shorter interval always means that the status and physical function of patients are poor and organ function has not recovered from the toxicity and immunosuppression of the conditioning regimens, patients cannot tolerate the conditioning regimens of a second HSCT^19–21^. In our study, 5 pediatric patients with primary GF underwent HSCT-2 within 60 days. There were 2 pediatric patients diagnosed as PGF. After transfusing mesenchymal cells and blood products intermittently, the hemogram recovered, and the donor chimerism was over 90%.

Overall, for SAA pediatric patients who suffer from GF without MSDs or MUDs, haploidentical-matched family donor HSCT is an effective treatment. However, there are no standard conditioning regimens. We also found that for SAA pediatric patients who use haploidentical-matched family donors, adding a low dose of Bu or 2 ~ 3 Gy TBI in NMA will not prolong the time of engraftment or increase the probability of acute or chronic GVHD. In addition, although the result showed that using PBSCs as a graft source are better for the engraftment of neutrophils, the samples was too small to verify our conclusion. We hope it can be discussed in future studies.
